# A Retrospective Analysis of the Demographics, Treatment, and Survival Outcomes of Patients with Desmoplastic Nodular Medulloblastoma Using the Surveillance, Epidemiology, and End Results (SEER) Database

**DOI:** 10.7759/cureus.9042

**Published:** 2020-07-07

**Authors:** Julian L Gendreau, Shishir Gupta, Tyler X Giles, Courtney E Stone, Mickey E Abraham, James G Lindley

**Affiliations:** 1 Medicine, Eisenhower Army Medical Center, Fort Gordon, Augusta, USA; 2 Medicine, Rollins School of Public Health, Emory University School of Medicine, Atlanta, USA; 3 Medicine, Mercer University School of Medicine, Macon, USA; 4 Medicine, Mercer University School of Medicine, Savannah, USA; 5 Neurosurgery, University of California, San Diego, USA; 6 Neurosurgery, Neurological & Spine Institute, Savannah, USA

**Keywords:** desmoplastic nodular medulloblastoma, epidemiology, medulloblastoma, radiotherapy, seer, survival, chemotherapy

## Abstract

Objective

Medulloblastoma is the most common malignant brain tumor in children younger than four years of age. Children diagnosed with desmoplastic nodular medulloblastoma (DNMB) have more favorable survival outcomes when compared to other subtypes of this disease and, to date, the demographics of DNMB have only been characterized by a few small clinical case series. Additionally, the current effort is being made at reducing radiotherapeutic modalities in this patient population to avoid the adverse effects associated with radiotherapy in children. Therefore, the goal of this study was to characterize the demographics, treatments, and survival outcomes of patients with DNMB using a large federal database.

Methods

The Surveillance, Epidemiology, and End Results database was queried to retrieve demographical, treatment, and survival data for patients diagnosed with DNMB. Statistical testing was performed with the R software stats package (R Foundation for Statistical Computing, Vienna, Austria). Student’s t tests and analysis of variance tests were used to measure differences among survival rates.

Results

Data from 360 patients with DNMB were retrieved from 1975-2016. There was a higher prevalence of DNMB in children younger than four years of age (33% of all cases). Males had a higher prevalence than females (57%). There was a preponderance of diagnoses in white individuals (82% of all cases) and more diagnoses in the Pacific Coast region (49% of all cases). Distant metastases were present at initial diagnosis in 8.7%. Surgery was performed in almost all patients, and gross total resection was achieved in 77%. The overall rate of survival was 77.8% at five years; age, sex, race, and geographical region of diagnosis were not associated with differences in survival outcomes. Patients with no radiotherapy had a lower rate of survival compared to patients with postoperative radiotherapy (mean difference = 19.7%; [95% CI 1.4%-38.0%], p = 0.0314). However, radiotherapy did not improve survival outcomes in patients undergoing chemotherapeutic treatment to a degree with any statistical significance. There was no statistically significant improvement in survival for patients undergoing radiotherapy prior to procedure when compared to patients with no radiotherapy.

Conclusions

In patients undergoing chemotherapeutic treatment for the DNMB subtype of medulloblastoma specifically, additional radiotherapy may offer only minimal benefit to the survival outcome. It is essential continued clinical trials be performed for the purpose of devising alternate treatments to radiotherapy.

## Introduction

Medulloblastoma is the most common malignant brain tumor in children younger than four years of age [1]. The WHO has divided medulloblastoma into four different histological subtypes, of which, desmoplastic nodular medulloblastoma (DNMB) is associated with the most favorable prognosis [2]. The WHO utilizes both histological and molecular profiling methods to identify and differentiate among these medulloblastoma subtypes. DNMBs are found to be hedgehog activated and TP53 wild-type [2].

Traditional approaches to treatment for medulloblastoma involve resection, chemotherapy, and craniospinal radiation for optimal survival outcomes [3]. Unfortunately, radiotherapy has been found to cause many long-term and detrimental side effects in children, including cognitive damage, central endocrinopathies, vasculopathies, as well as having been associated with the induction of secondary malignancies post-therapy [4-6]. Therefore, current effort is being made in identifying and investigating alternative treatment options to radiotherapy that also offer equal survival outcomes. To date, the use of post-operative chemotherapy comprising methotrexate and autologous hematopoietic cell rescue has been found to offer promising rates of survival without radiotherapy [7-10]. Investigating alternative treatment options for the purpose of minimizing the side effects of radiotherapy is crucial for DNMB since these patients already have more favorable rates of survival when compared to other medulloblastoma subtypes.

One goal of this study was to further characterize outcomes associated with radiotherapy use in this patient population. Additionally, there are only a few clinical case series describing patients with the DNMB subtype of medulloblastoma specifically [11-13]. Therefore, another goal of this study was to characterize the demographics of this patient population using a large patient size from a longitudinal database. To accomplish these goals, the Surveillance, Epidemiology, and End Results (SEER) database was queried during the time frame of 1975-2016 for all patients diagnosed with DNMB. All data of this patient population’s demographics, treatments, and rates of survival were extracted and statistically analysed.

## Materials and methods

Database information

The SEER database is a federally funded cancer data repository made available by the United States Centers for Disease Control and Prevention, the United States National Cancer Institute, and several other regional and state cancer registries [14]. It is a well-validated system for providing individual patient data for the purpose of measuring the incidence, prevalence, mortality rates, and treatment modalities of cancer in the United States [15].

Database search parameters

The SEER database was queried for all patient data of individuals diagnosed with DNMB using guidelines previously suggested for analysing large-volume databases [16]. Data for demographic characterization was retrieved from the most recent dataset: Incidence - SEER 18 Regs Research Data + Hurricane Katrina Impacted Louisiana Cases, Nov 2018 Sub (1975-2016 varying). For the purpose of retrieving data on survival outcomes, the following database was used: Incidence - SEER 18 Regs Research Data + Hurricane Katrina Impacted Louisiana Cases, Nov 2018 Sub (2000-2016) <Katrina/Rita Population Adjustment>. For the purpose of retrieving chemotherapeutic and radiotherapeutic data, the following dataset was used: Incidence - SEER 9 Regs Custom Data (with additional treatment fields), Nov 2018 Sub (1975-2016) <Katrina/Rita Population Adjustment>. The data were extracted and survival rates were calculated with the SEER*STAT v 8.3.6 software (National Cancer Institute, Bethesda, MD). All patients with the International Classification of Diseases for Oncology, Third Edition (ICD-O-3) diagnosis code of 9471/3 for DNMB were included.

Collected data included sex, race, geographical region, age at diagnosis, metastasis at diagnosis, extent of surgical resection, treatment with chemotherapy, treatment with radiotherapy, and sequence of radiotherapy as it related to the surgical resection of the tumor. Survival outcomes were also calculated at one, two, three, four, and five years post diagnosis.

The extent of surgical resection was divided into gross total resection (GTR) and subtotal resection (STR). The GTR group had patients identified with surgical codes 55 (GTR of the lobe of brain), 40 (partial resection of the lobe of brain), and 30 (radical, total, gross resection of tumor, lesion mass, or mass in brain). The STR group was identified with patients who were listed with surgical codes 27 (excisional biopsy), 22 (partial resection of tumor, lesion, or mass), 21 (STR of tumor, lesion, or mass, not otherwise specified [NOS]), and 20 (local excision of tumor, lesion, or mass, excisional biopsy, or stereotactic biopsy of the brain).

Metastases at diagnosis data included the diagnosis codes of 50 (distant metastasis in addition to a distant metastasis at a lymph node), 20 (distant metastasis at a lymph node plus a metastasis at an inferior vena cava lymph node), 10 (metastasis present at distant lymph nodes, NOS), and 0 (no metastases present at diagnosis).

Statistical analysis

Analysis and visualization were performed with the R software (R Foundation for Statistical Computing, Vienna, Austria) using the stats package. The analysis was performed according to guidelines established for describing large observational datasets.Student’s t tests were used to calculate mean differences and 95% confidence intervals when comparing two separate means. Analysis of variance (ANOVA) testing was used to compare means across multiple groups. Subsequent post hoc analysis was performed with Tukey’s test. In this statistical testing, p < 0.05 was considered significant.

## Results

Demographics

There were 360 patients with DNMB diagnosed from 1975 to 2016. Males had a higher prevalence than female patients (57%). There were 33% of the total DNMB diagnoses in children younger than four years of age. There was a preponderance of diagnoses in white individuals (82% of all cases) and individuals in the Pacific Coast geographical region (49% of all cases). Surgery was performed in 96.7% of patients, and GTR was achieved in 77%. Patient demographics including sex, race, geographic region, age, and extent of resection are displayed in Table 1.

**Table 1:** Demographics of patients diagnosed with DNMB DNMB = desmoplastic nodular medulloblastoma; GTR = gross total resection; STR = subtotal resection. *American Indian, Asian/Pacific Islander.

Characteristic		N	% of demographic
Overall		360	-
Sex	Male	206	57
	Female	154	43
Race	White	295	82
	African American	28	8
	Other*	37	10
Region	Alaska	0	0
	East	110	31
	Northern Plains	34	9
	Pacific Coast	176	49
	Southwest	40	11
Age	<1	20	6
	1-4	100	28
	5-9	56	16
	10-14	26	7
	15-19	29	8
	20-24	30	8
	25-29	28	8
	30-34	24	7
	35-39	18	5
	40-44	8	2
	45-49	9	2
	50-54	8	2
	>55	4	1
Resection	GTR	216	77
	STR	66	23

Tumor size ranged from 12 to 73 mm. Laterality was largely equal with most tumors labeled as “not a paired site.” Of the tumors where laterality was specified, 47% were identified as right-sided origin and 53% were identified as left-sided origin. 

Distant metastases were present at initial diagnosis in 8.7% patients. Locations of metastatic disease at initial diagnosis included the bone (one case), other areas of the brain (four cases), and at unspecified locations (two cases).

Survival analysis

Survival data was available for 270 patients with data ranging from 2000 to 2016. Survival data was not collected before 2000 for this particular SEER database. Overall survival at five years was 77.8%. Rates of survival at five years stratified by demographical subgroups are displayed in Table 2.

**Table 2:** Results of survival analysis in patients with DNMB DNMB = desmoplastic nodular medulloblastoma.

Characteristic		N	Five-year survival rate %
Overall		270	77.8
Sex	Male	159	80.5
	Female	111	73.7
Race	White	219	78.7
	African American	20	85.1
	Asian or Pacific Islander	28	67.1
	American Indian/Alaska Native	3	66.7
Region	East	87	73.4
	Northern Plains	20	79.1
	Pacific Coast	139	80.5
	Southwest	24	77.4
Age	<1	15	100
	1-4	83	75.6
	5-9	43	83.9
	10-14	19	78.6
	15-19	25	71.6
	20-24	23	79
	25-29	24	72
	30-34	14	71.4
	35-39	9	84.6
	40-44	4	100
	45-49	5	36
	50-54	5	75
	>55	2	50

Student’s t testing revealed no statistically significant differences in survival between sex categories. ANOVA revealed no statistically significant variances among race, geographical region, or age groups associated with survival outcomes.

Radiotherapy and chemotherapy

The database included data for 169 patients regarding their status of undergoing either treatment with chemotherapy or radiotherapy (Table 3).

**Table 3:** Number of patients stratified by radiotherapy sequence with surgery

	No radiotherapy (%)	Radiation prior to surgery (%)	Radiation after surgery (%)
No chemotherapy	15 (88.8)	4 (2.4)	34 (20.1)
Chemotherapy	39 (23.1)	1 (0.6)	76 (45.0)
Total	54 (32.0)	5 (3.0)	110 (65.1)

Chemotherapy was reported using binary values only: either with or without chemotherapy. Patients who underwent chemotherapy had a higher rate of survival at five years than patients who did not undergo chemotherapy (mean difference = 20.4%; [95% CI 5.2%-35.6%], p < 0.01).

Radiotherapy was reported as binary variables: with beam radiotherapy or without beam therapy. Entries involving radioactive implants (two), refused radiotherapy (two), and unknown if administered (one) were excluded from the analysis. Radiotherapy sequence was reported as eight different potential variables: no radiation delivered, radiation prior to surgery, radiation after surgery, radiation both before and after surgery, intraoperative radiation, intraoperative radiation with either additional radiotherapy before or after surgery, surgery both before and after radiation, and unknown sequence of radiotherapy with surgery. Only the treatment categories of no radiation, radiation prior to surgery, and radiation after surgery had entries of patient data.

**Figure 1:**
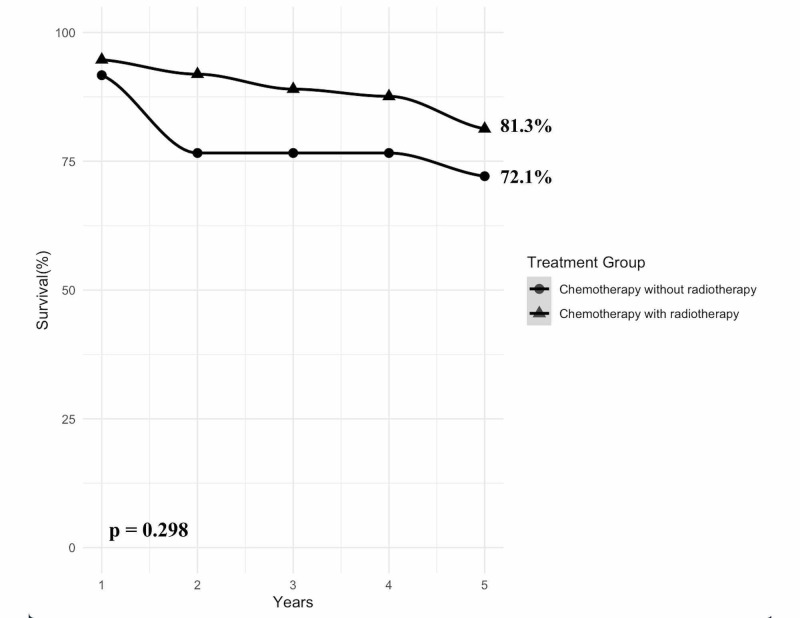
Survival of DNMB patients undergoing chemotherapy with and without radiotherapy DNMB = desmoplastic nodular medulloblastoma.

Patients undergoing beam radiation had a statistically significant improved survival rate when compared to patients not undergoing radiotherapy (mean difference = 16.4%; [95% CI 0.54%-32.34%], p = 0.043). Of patients undergoing chemotherapeutic treatment (N = 116), radiotherapy did not have a statistically significant improvement in survival outcome at five years (Figure 1).

**Figure 2:**
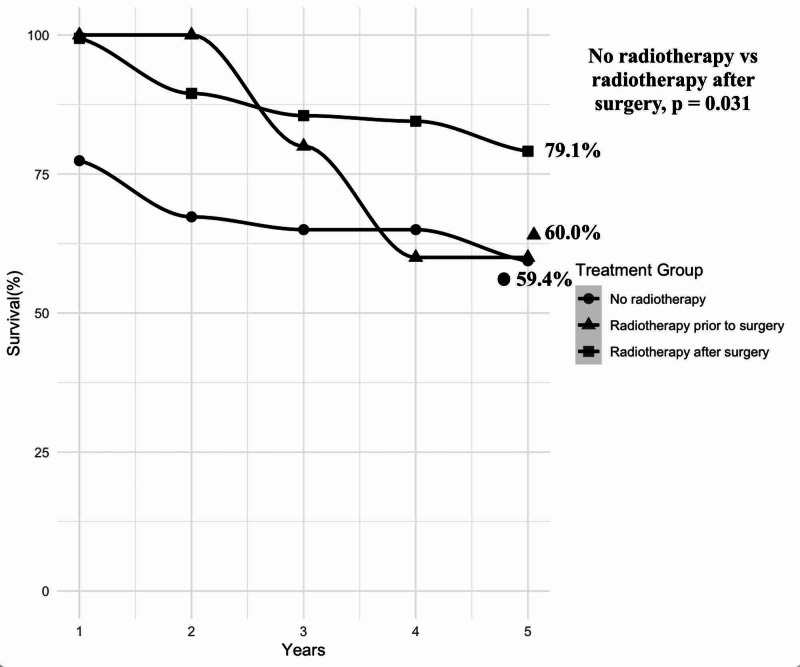
Survival of DNMB patients stratified by radiotherapy sequence with surgery DNMB = desmoplastic nodular medulloblastoma.

ANOVA revealed variances among radiotherapy sequence and survival (p < 0.035). Post hoc Tukey’s testing revealed that patients undergoing no radiotherapy had a lower rate of survival at five years when compared to patients with radiotherapy after surgery (mean difference = 19.7; [95% CI 1.4%-38.0%], p = 0.031). There was no statistical difference between patients undergoing no radiotherapy and radiotherapy prior to surgery. Survival plot of radiotherapy sequence as it relates to surgery is displayed in Figure 2.

## Discussion

The SEER database includes approximately 28% of the US population, and this retrospective analysis retrieved data on 360 patients diagnosed with DNMB in the database. Therefore, it is reasonable to assume that the total incidence of DNMB was approximately 1286 diagnoses spanning the time frame of 1975-2016 in the United States. It is also important to consider that the database included a “medulloblastoma not otherwise specified” classification, which could have potentially contained additional diagnoses of DNMB. Thus, this calculation could underestimate the total incidence during this time frame.

In terms of demographics, when comparing this study to a recent retrospective analysis of all medulloblastoma diagnoses in the SEER database by Khanna et al., DNMBs appear to be diagnosed in patients in younger age ranges [17]. In the age group ranges of younger than one year to one through four, the percentage increased significantly (3.08% to 21.37%) for all medulloblastomas, and it likewise did in this study (5.56% to 27.78%). However, in all medulloblastomas, the prevalence again increased from the one- to four-year to the five to nine age groups (21.37% to 25.68%), and in this study the prevalence decreased between these age groups (27.78% to 15.56%). Demographical percentages of sex and race were similar between both database reviews. Of note, there was original evidence to suggest that DNMBs were predominantly found in younger patients by a case series of 12 patients, which is supported by this study [18]. However, this same case series also suggested a female predominant distribution of DNMB, which was not supported by this present database review (males 57%; females 43%).

In the study by Khanna et al., overall survival was much lower for all medulloblastomas when comparing to the data for the DNMB subtype specifically. In all medulloblastomas, the survival rate for the age group of younger than one year was 48.4%. For DNMBs, this review found 100% survival rates, providing further evidence that this subtype offers better prognoses for patients in terms of survival. In the one- to four-year age group, survival in medulloblastomas was 61.9% compared to 75.6% in DNMBs [17]. It is thought that patients younger than three years of age had poorer prognoses for all medulloblastomas; however, this may not be true for the DNMB subtype [19,20]. When considering differences in sex, it was found that survival outcomes were better in females by a previous study when analysing all medulloblastomas, but for DNMB, the survival outcome appears slightly better in males (males 80.5%; females 73.7%) [21].

Before this database review, one of the largest studies measuring survival rates for the DNMB subtype specifically was a multicenter retrospective review including 108 patients. All individuals underwent chemotherapies, radiotherapies, and autologous hematopoietic stem-cell transplantations according to their prospective clinical trials for which they were enrolled. In patients with early-childhood DNMB, a survival rate of 72% was found at eight years in 87 patients [7]. The rates provided by this study are similar to the survival rates found in this database review when measuring survival at five years (100% survival, age less than one; 75.6% survival, age one to four years).

When considering eliminating radiotherapy for medulloblastomas, Rutkowski et al. found that patients undergoing chemotherapy with persistent post-chemotherapeutic surveillance had comparable survival to patients undergoing both chemotherapy and radiotherapy when looking at early childhood medulloblastomas (10-year survival = 63.6% and 55.2%, respectively, p = 0.274) by comparing results from the HIT-SKK’87 (Therapieprotokoll für Säuglinge und Kleinkinder mit Hirntumoren [Brain Tumor Radiotherapy for Infants and Toddlers with Medulloblastoma] 1987) and HIT-SKK’92 trials [22]. This overall survival rate is lower than the rates in this analysis, which is to be expected as these trials included all subtypes of medulloblastomas. Many other reviews have found that avoiding radiotherapy is a viable option for young children without metastatic disease and for children with complete resection [8,23,24]. Apart from these data, there are several other clinical trials that have been performed measuring the impact of avoidance of radiotherapy on survival outcome for other tumors of the central nervous system [25,26]. These trials have shown promising results and are pioneering the future in avoiding the long-term cognitive deficits associated with radiotherapy. Avoiding the need for radiotherapy is also beneficial to patients in resource-limited areas that are unable to offer these types of advanced treatment modalities [27].

For the DMNB subtype of medulloblastoma tumors specifically, there are even more promising outcomes. In the HIT-SKK protocol trials, the authors used intravenous and intraventricular methotrexate in place of radiotherapy except for patients with metastatic disease or residual disease after chemotherapy. In their long-term data of eight years, overall survival was reported to be 91% in 115 patients [28]. Another previous review of HIT protocol trials also supports this good prognosis in DNMB patients [29]. Despite this, there were concerns of poor neurocognitive outcomes with the intraventricular methotrexate. After these concerns, a new clinical trial with 26 participants diagnosed with DNMB was developed by the Children’s Oncology Group eliminating this intraventricular methotrexate (clinical trial #NCT02017964), but it was aborted early in 2018 when the progression-free survival dropped lower than 90%.

Recently in 2020, Dhall et al. reported excellent survival rates in 92 DNMB patients enrolled in the prospective multicenter “Head Start” III trial using myeloablative chemotherapy and autologous hematopoietic cell rescue. They reported preservation of mean intelligence quotient scores and quality of life scores with an overall survival rate of 89% at five years [30]. Interestingly, this database review found that patients undergoing chemotherapeutic treatment did not have improvement in five-year survival outcomes to any statistical significance (81.3% vs 72.1%, p = 0.298) among 116 patients. Therefore, this review provides further evidence that adding radiotherapeutic treatment to patients undergoing chemotherapy for the DNMB subtype of medulloblastoma may have only minimal benefit to survival outcomes.

Limitations

This database offered no metrics to measure intelligence scores or quality of life scores in patients who underwent these regimens of surgeries, chemotherapies, and radiotherapies. Neither did this database measure cognitive changes in children undergoing long-term radiotherapy, measure rates of seizure control, or measure rates of cranial neuropathies associated with these diseases. All of these outcomes should be characterized with future clinical case series.

This database is currently unable to monitor the molecular profiles of these tumors. Therefore, correlations of specific molecular profiles to outcomes post-intervention were unable to be made. Future updates should further optimize and adapt the database to store such molecular profiling for cancer data in future.

Some patient age ranges included a small number of individual cases (40-45, 45-49, 50-54 had five patients; 60-64, 65-69 had one patient). This limited the statistical power of this analysis when looking at the data of DNMB, especially in adult diagnoses.

It is also important to consider that the database included a “medulloblastoma not otherwise specified” classification, which could have potentially contained additional diagnoses of DNMB that went unreviewed in this study and were not included in the final data or conclusions.

## Conclusions

With this retrospective review of 360 patients diagnosed with DNMBs, the authors find that DNMBs follow trends similar to all medulloblastomas except for having an increased percentage of diagnoses in the younger than four age range. From 1975 to 2016, a majority of the patients underwent resection of the tumor, and received chemotherapy, as well as post-operative radiotherapy. In patients undergoing chemotherapeutic treatment for DNMB, additional radiotherapy was found to offer only minimal benefit to the survival outcome that did not achieve statistical significance. This subtype of medulloblastoma has a favorable survival outcome, but patients still experience significant long-term morbidity as a result of these lifesaving treatments. Devising therapeutic modalities toward the reduction of these long-term adverse effects related to treatment is an important goal for the neurosurgical community.
